# Analyses of circRNA Expression throughout the Light-Dark Cycle Reveal a Strong Regulation of *Cdr1as*, Associated with Light Entrainment in the SCN

**DOI:** 10.3390/ijms232012347

**Published:** 2022-10-15

**Authors:** Andranik Ivanov, Daniele Mattei, Kathrin Radscheit, Anne-Claire Compagnion, Jan Patrick Pett, Hanspeter Herzel, Rosa Chiara Paolicelli, Monika Piwecka, Urs Meyer, Dieter Beule

**Affiliations:** 1 Core Unit Bioinformatics, Berlin Institute of Health, Charité-Universitätsmedizin Berlin, 10117 Berlin, Germany; 2 MSSM Department of Neuroscience, Icahn School of Medicine at Mount Sinai, New York, NY 10029, USA; 3 Max-Delbrück-Center for Molecular Medicine, 13125 Berlin, Germany; 4 Charité-Universitätsmedizin Berlin, Einstein Center for Neurosciences Berlin, 10117 Berlin, Germany; 5 Department of Biomedical Sciences, University of Lausanne, 1005 Lausanne, Switzerland; 6 Cellular Genetics Programme, Wellcome Sanger Institute, Hinxton, Cambridge CB10 1SA, UK; 7 Institute for Theoretical Biology, Charité-Universitätsmedizin Berlin, 10117 Berlin, Germany; 8 Department of Non-Coding RNAs, Institute of Bioorganic Chemistry, Polish Academy of Sciences, 61-704 Poznan, Poland; 9 Institute of Pharmacology and Toxicology, University of Zurich-Vetsuisse, 8057 Zurich, Switzerland

**Keywords:** circular RNA (circRNA), *Cdr1as*, circadian rhythm, suprachiasmatic nucleus, SCN, non-coding RNA, *Cyrano*, light entrainment, miR-7, light-dark, wake-sleep

## Abstract

Circular RNAs (circRNAs) are a large class of relatively stable RNA molecules that are highly expressed in animal brains. Many circRNAs have been associated with CNS disorders accompanied by an aberrant wake-sleep cycle. However, the regulation of circRNAs in brain homeostasis over daily light-dark (LD) cycles has not been characterized. Here, we aim to quantify the daily expression changes of circRNAs in physiological conditions in healthy adult animals. Using newly generated and public RNA-Seq data, we monitored circRNA expression throughout the 12:12 h LD cycle in various mouse brain regions. We identified that *Cdr1as*, a conserved circRNA that regulates synaptic transmission, is highly expressed in the suprachiasmatic nucleus (SCN), the master circadian pacemaker. Despite its high stability, *Cdr1as* has a very dynamic expression in the SCN throughout the LD cycle, as well as a significant regulation in the hippocampus following the entry into the dark phase. Computational integration of different public datasets predicted that *Cdr1as* is important for regulating light entrainment in the SCN. We hypothesize that the expression changes of *Cdr1as* in the SCN, particularly during the dark phase, are associated with light-induced phase shifts. Importantly, our work revises the current beliefs about natural circRNA stability and suggests that the time component must be considered when studying circRNA regulation.

## 1. Introduction

Circadian rhythms are physiological, molecular, and behavioral changes that follow a 24-h cycle. Most multicellular organisms take advantage of environmental factors, such as light and temperature, to fine-tune their daily behavior and metabolism to create a coherent circadian system. In mammals, the suprachiasmatic nucleus (SCN) is the central circadian pacemaker [1,2] that is entrained in light-dark (LD) cycles [3]. From the retina, the light signal is transmitted to the SCN, which then synchronizes clocks in other tissues [4]. The endogenous mammalian circadian clock consists of transcription-translation feedback loops [5] that are accompanied by global changes in transcriptional and post-transcriptional regulation [6,7], including RNA synthesis and degradation, alternative splicing, and editing [8,9,10,11]. As of now, it is clear that many RNA species, both protein-coding and non-coding, play an essential role in maintaining the circadian rhythm [6,7,12,13]. However, circular RNAs (circRNAs), an alternative form of RNA splicing products, dependent on temperature and RNA editing [14,15,16,17], have not been well studied throughout daily activities and circadian rhythm.

CircRNAs are a large class of highly stable RNA molecules that consist of multiple or single exons [18,19,20]. They have conserved biogenesis and are expressed in animals and plants [14,16,18,19,21]. CircRNAs are primarily localized to the cytoplasm and have various functions, such as sequestering miRNAs, modulation of RNA stability, and multiple interactions with RNA-binding proteins [16,18,22,23]. A subset of circRNAs with retained introns (so-called exon-intron circRNAs) reside in the nucleus and regulate mRNA transcription [24]. Certain circRNAs can be translated into proteins [25,26] or released from cells in extracellular vesicles [27,28]. In animals, circRNAs are highly expressed in the brain, and a subset of circRNAs is enriched at the synaptic terminals [15,29]. Due to their high stability and synaptic enrichment, these molecules are thought to be involved in intracellular information transport, long-term memory formation, and memory consolidation [30]. Moreover, the deregulation of circRNAs has been implicated in neurodegenerative, psychiatric, and neurodevelopmental disorders [31,32,33,34,35]. Many of these brain conditions occur in parallel, or arguably due to aberrant wake/sleep cycles [36,37], which is why it is necessary to study circRNAs in the context of the circadian rhythm. Finally, due to their relatively stable nature and tissue-specific expression pattern, circRNAs have been recognized as biomarkers in different pathologies [38], particularly in many cancer types [39], where circRNAs are systematically deregulated [40]. Given the recent observations that cancer metastases can accelerate during the rest (sleep) phase [41], it becomes evident that the regulation of circRNAs should be analyzed in both the wake and sleep phases. Here, we studied circRNA expression in different mouse brain regions during 12:12 h LD cycles and predicted *Cdr1as* as an essential regulatory molecule that impacts the light entrainment in the SCN. *Cdr1as* is highly expressed in the brain, particularly in glutamatergic neurons [31]. It has an unusually high number of miR-7 binding sites [19,22]. MiR-7 is a potent, brain-enriched miRNA [42,43] that has been implicated in the pathogenesis of different brain diseases [44,45,46,47,48,49,50,51,52]. *Cdr1as* and miR-7 regulate neuronal activity through a network with two other non-coding RNAs, miR-671 and *Cyrano* [53]. The loss of *Cdr1as* in mice results in the deregulation of excitatory synaptic transmission and the upregulation of immediate early genes [31]. *Cdr1as* deletion in mice also leads to vision defects [54]. Our findings explain previously observed gene expression changes and phenotypes in *Cdr1as* mutant mice and add another piece to the puzzle of *Cdr1as* functions. 

## 2. Results

### 2.1. Cdr1as Is Highly Expressed in the SCN and Has a Significant Differential Expression during the 12:12 h Light-Dark Cycle

**Figure 1:**
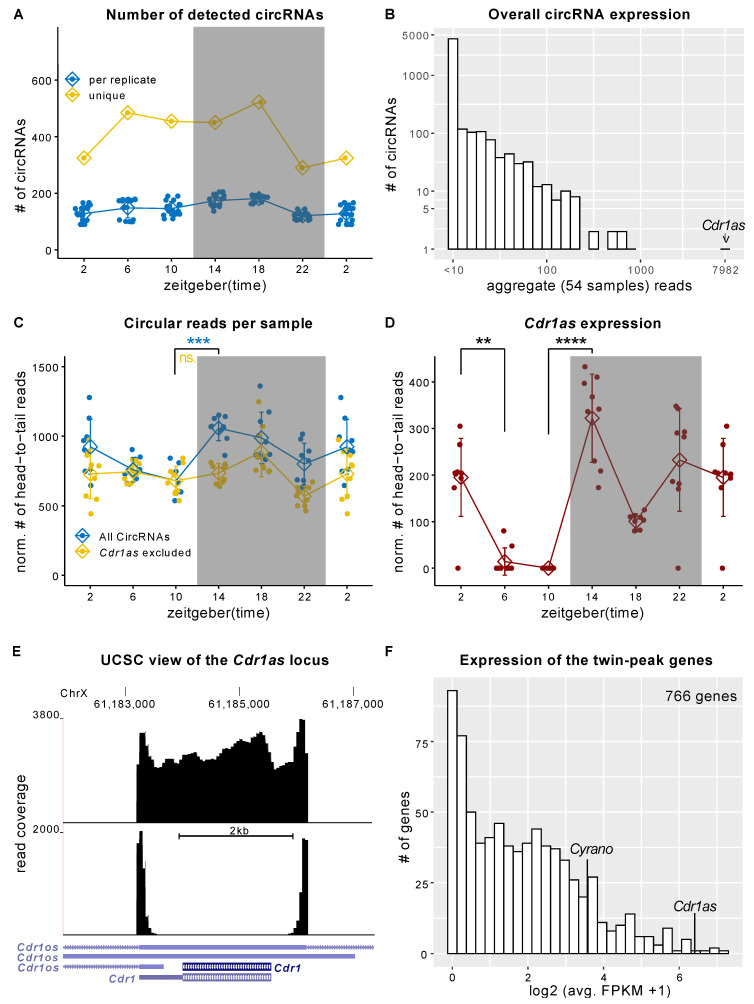
*Cdr1as* expression changes during the 24 h LD cycle in the SCN. (**A**) The number of detected circRNAs for each replicate and each time point. (**B**) Overall expression of circRNAs. For each circRNA, we summed up circularization-supporting head-to-tail reads in all samples/groups. *Cdr1as* has an order of magnitude higher expression than any other circRNA. (**C**) Normalized (to the number of mapped reads) circular reads in the 12:12 LD cycle. Error bars show the SD. (Bonferroni) Adjusted *t*-test *p*-value: *** < 10^−5^, ns is not significant (**D**) Normalized expression of *Cdr1as* circRNA during the 12:12 LD cycle. Error bars show SD. DESeq-2 adj. *p*-value: ** < 10^−3^. **** < 10^−16^. (**A**–**D**) use periodic continuation plotting at ZT2. (**E**) UCSC genome browser view of the *Cdr1* locus using a pool of reads from three replicates at ZT14. The lower panel shows only circular (head-to-tail) reads. The upper panel shows all reads mapped to the locus. (**F**) Histogram showing the expression (FPKM) of all 766 twin-peak module genes from Pembroke et al. [7].

We quantified the circRNA expression levels in the SCN by systematically analyzing the total RNA-Seq data from Pembroke et al. [7]. The authors monitored six equidistant time points in nine replicates during a 12:12 h LD cycle. We identified a total of 1691 expressed circular RNAs in the SCN. The number of consistently detectable circRNAs was highest in the middle of the dark phase, with a substantial drop at its end (325, 485, 455, 450, 523, and 290 circRNAs at ZT2, ZT6, ZT10, ZT14, ZT18, and ZT22, respectively; cf. Figure 1A).

*Cdr1as* circRNA showed the highest expression of all (Figure 1B, Supplementary Table S1). It was expressed one order of magnitude more than any other circRNA in the SCN (Figure 1B). *Cdr1as* contributed the most to the overall differences in circular reads during the whole 12:12h LD cycle (Figure 1C,D) and was the only highly expressed circRNA with significant regulation in SCN over the LD cycle (e.g., ZT14 vs. ZT10: DESeq2 adj. *p*-value 1.3 × 10^−19^). A rather unexpected observation is that this stable circRNA is downregulated more than twofold in four hours (Figure 1D) and again upregulated, exhibiting twin-peak expression with peaks at the beginning and the end of the dark cycle (Figure 1D). Interestingly, Pembroke et al. [7] described a cluster of 766 genes with exact twin-peak expression as a synaptic module regulating light-induced phase shifts in the SCN. Inspecting the Cdr1 locus in the UCSC genome browser showed all reads mapping to the *Cdr1* locus map within the circRNA boundaries rather than the primary transcript (Figure 1E). Thus, all reads mapping to the Cdr1 gene could be attributed to the circular RNA, allowing a better *Cdr1as* expression quantification (Supplementary Figure S1) and putting *Cdr1as* into the top 5 highest (sorted by FPKM) expressed RNAs in the synaptic twin-peak module described by Pembroke et al. (Figure 1F). Variability between replicates differs substantially between time points. It is highest near the light-dark transitions (Supplementary Figure S1, Figure 1D), which might hint at quick regulatory processes. Interestingly, the long non-coding RNA *Cyrano*, known to serve as a negative regulator of miR-7 in the brain [53], showed the same twin-peak expression pattern as *Cdr1as* (Supplementary Figure S1, Figure 1F).

### 2.2. The Expression of Cdr1as-Associated Genes Significantly Depends on Light Induction

**Figure 2:**
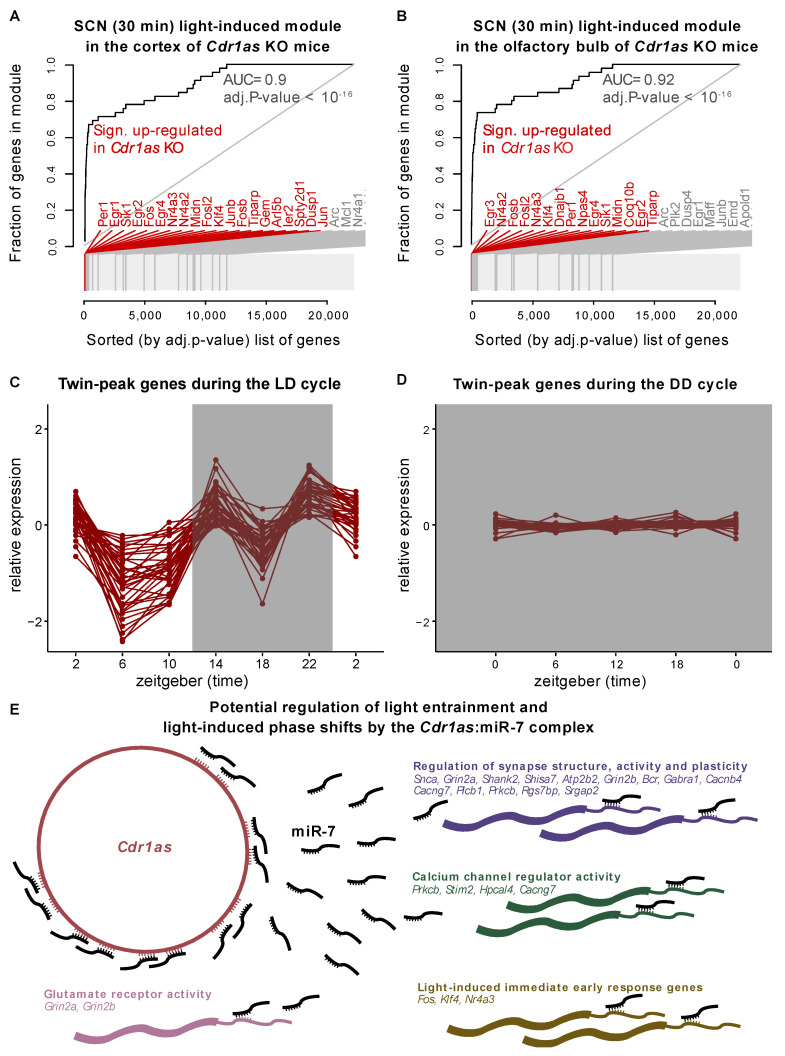
The impact of light on the expression of *Cdr1as* and associated genes. Evidence plots show enrichment of SCN immediate light-induced genes (defined in Xu et al. [56]) in the (**A**) cortex and (**B**) olfactory bulb of *Cdr1as* KO mice. The X-axis shows the list of sorted genes (by adj. *p*-value *Cdr1as* KO vs. WT) from polyA RNA-Seq in Piwecka et al. [31]. Y-axis is the fraction of the gene set defined by Xu et al., 2021 [56]. In red are genes upregulated in *Cdr1as* KO with adj. *p*-value < 0.05. (**C**) Log2 expression (normalized to the temporal mean) of the top 50 genes (sorted by FPKM) from the twin-peak module in the LD cycle [7]. (**D**) The same set of genes (as in C) in the DD cycle. Cheng et al. [57] measured RNA expression in control and Sox2 mutant mice in the DD cycle at four different time points. Here, we used only control samples. (**E**) *Cdr1as* and miR-7 are both expressed in the SCN. Predicted targets of miR-7 were downloaded from Targetscan [60]. Twin-peak genes are defined in Pembroke et al. [7]. The immediate light-induced gene module is from Xu et al. [56]. The annotation of other pathway modules is from the MsigDB database.

Piwecka et al. [31] showed that loss of *Cdr1as* results in the upregulation of certain circadian genes (*Per1*, *Sik1*, *Klf10*, *Npas4*) and upregulation of immediate early genes, such as *Fos*, *Egr1*, *Klf4*, *Jun*, etc. This group of immediate early genes is activated and rapidly transcribed in the SCN upon photic stimuli [55]. Recently, the Takahashi lab measured genome-wide mRNA changes in the SCN after a short period of light exposure (e.g., 30 min) [56]. This early light-induced gene set is significantly enriched in genes deregulated upon *Cdr1as* KO in different brain regions (Figure 2A,B). Further, Xu et al. [56] identified that the *Npas4* transcription factor is an essential regulator of circadian behavior and transcriptional response to light in the SCN [56]. In the polyA+ RNA-Seq of the *Cdr1as* KO mice [31], *NPas4* is significantly upregulated in three (hippocampus, cerebellum, olfactory bulb) out of the four sequenced brain regions. Moreover, in the hippocampus (of *Cdr1as* KO mice), *Npas4* is the 11th and the cerebellum’s 7th most significantly deregulated gene. 

To further study the effect of light on *Cdr1as* regulation, we analyzed the expression of twin-peak cluster genes (defined by Pembroke et al.) in the SCN during constant darkness, using data from Cheng et al. [57]. The study provides RNA-Seq at four circadian time points (CT = 0, 6, 12, 18) in murine SCN with five replicates. Before harvesting, the mice were released from the standard 12:12 h LD cycle into complete darkness for two days (dark-dark or DD cycle). We selected the top 50 highest expressed genes from the twin-peak module and monitored their expression in LD and DD cycles. Due to the polyA enrichment protocol used by Cheng et al., *Cdr1as* circRNA itself is not detectable in this data. However, none of the 49 other highly expressed genes changed their expression significantly during the DD cycle (Figure 2C,D). Our observations hold when the analysis is carried out using the top 100 or top 200 highest expressed genes (sorted by FPKM) from the twin-peak module (Supplementary Figure S2). This result suggests that the overall expression of the twin-peak cluster and likely *Cdr1as* in murine SCN highly depends on light exposure. 

Light is communicated to the SCN from the retina by glutamatergic neurotransmission from the retinohypothalamic tract. Given the *Cdr1as* enrichment specifically in glutamatergic neurons [31] and high expression of miR-7 in the SCN [58,59], we suggest that *Cdr1as* may be an important regulator of glutamatergic synaptic transmission during light-induced phase shifts through the delivery of miR-7 to its early response and twin-peak target genes. The overlap of miR-7 targets (444 in total from the Targetscan database [60]) and twin-peak cluster (766) identified 37 genes (Supplementary Table S2) that could be regulated by *Cdr1as*/miR-7 complex in the context of light-induced phase-shifts. Pembroke et al. [7] reported that the twin-peak cluster is enriched in synaptic transmission and calcium signaling. As expected, its subset miR-7 targets are also significantly enriched in these biological pathways (Supplementary Table S2). Thus, it is conceivable that the *Cdr1as*:miR-7 complex plays a role in regulating photic inputs transmitted into the SCN (Figure 2E). Among miR-7 target genes from the twin-peak cluster that can be regulated by *Cdr1as*:miR-7 in the SCN, we can find ionotropic glutamate receptor complex (*Grin2a* and *Grin2b)*, calcium signaling genes (via *Prkcb*, *Stim2*, *Hpcal4*, and *Cacng7*), and genes involved in the regulation of the synaptic structure and its activity (*Snca*, *Grin2a*, *Shank2*, *Shisa7*, *Atp2b2*, *Grin2b*, *Bcr*, and *Gabra1)* (Figure 2E). The first response to light is likely regulated via *Fos*, *Klf4*, and *Nr4a3* (Figure 2E).

### 2.3. CircRNA Expression in the Hippocampus and Frontal Cortex Follows Constitutive Transcription Patterns

To further characterize the circadian expression of circRNAs, we sequenced total RNA from two additional brain regions: the hippocampus and frontal cortex. Mice were dissected at six equidistant time points throughout the LD phases. While the SCN acts as a circadian master pacemaker, other brain regions also display oscillatory capacity [61]. The hippocampus is particularly interesting, as it is essential for sleep-dependent memory consolidation [62]. It is interesting to study circRNA regulation in this context, as circRNAs are highly expressed in the hippocampus [15] and have a much longer lifetime than linear RNAs. Therefore, they may be involved in memory consolidation and the transport of information between different cell types [30]. We also investigated the frontal cortex, which is associated with many psychiatric and neurodegenerative disorders. 

**Figure 3:**
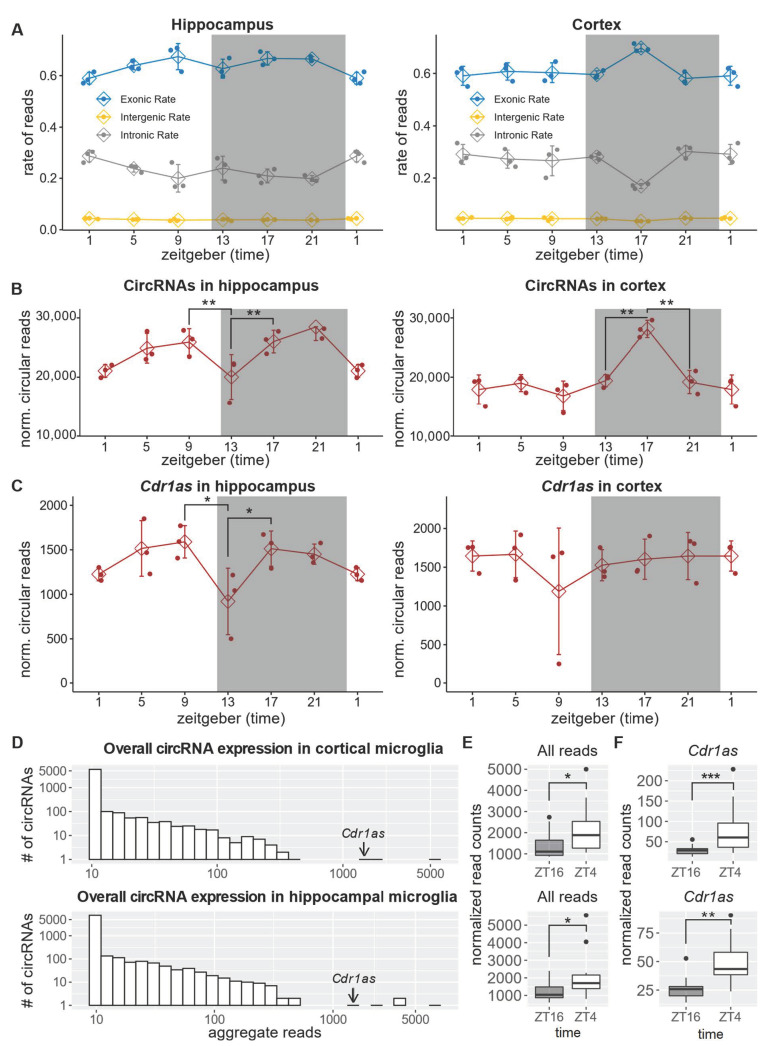
CircRNAs in the hippocampus and frontal cortex. (**A**) Genomic annotation of the sequencing reads. The X-axis is the (zeitgeber) time, and the Y-axis is the fraction of reads. (**B**) Normalized (norm. factor = total number of reads/avg. library size) number of circular reads per time point. Error bars show SD. (Bonferroni) Adjusted *p*-value: ** < 0.01. (**C**) Normalized (norm. factor = total number of reads/avg. library size) number of circular reads supporting *Cdr1as*. DESeq2 adj. *p*-value: * < 0.05 (**D**) Analogous to Figure 1B.(**E**) Normalized (norm. factor = total number of reads/avg. library size) number of all circular reads detected in hippocampal (**down**) and cortical (**up**) microglia sequencing. Each group represents a distribution of 16 replicates. (Bonferroni) Adjusted *T*-test *p*-value: * < 0.05. (**F**) *Cdr1as* expression (normalized circular reads) in microglia from the cortex (upper panel) and hippocampus (lower panel). Each box has 16 samples. DESeq2 adj. *p*-value: *** < 10^−5^, ** < 0.01.

Using three replicates at six time points (SE 150 bp, average sequencing depth 46 mil.), we identified 5505 and 4790 distinct circRNAs expressed in the hippocampus and frontal lobe (Supplementary Table S3). Our analysis of the frontal cortex identified 1328 of 1770 (75%) *circbase* [63] circRNAs, and in the hippocampus, 1297 of 1676 *circbase* circRNAs. In the hippocampus, along with a general reduction of exonic reads (Figure 3A) circRNA expression decreased by 20% during the transition from the dark to the light phase (Figure 3B). However, such a change in circularized RNA was most probably due to the difference in the expression of the linear host transcripts (Supplementary Figure S3). The expression of *Cdr1as* in the hippocampus was downregulated by 30% during the transition from the light to the dark phase and again upregulated during the dark phase. In the frontal cortex, *Cdr1as* expression did not change significantly during the LD cycle (Figure 3C). Resembling changes in exonic rates (Figure 3A), the overall circRNA expression in the cortex increased by 30% in the wake phase and was downregulated again just before the light phase started (Figure 3B). Similar to the hippocampus, such a fluctuation of circularization was due to a general change in the expression of the linear host gene (Supplementary Figure S3).

### 2.4. Cdr1as Is the Only Significantly Deregulated circRNA between Day and Night in Microglia

Microglia are brain-resident macrophages, and their inflammatory responses are controlled by the intrinsic circadian clock [64]. Microglia also exhibit morphological differences between wake and sleep, and disruption of the clock system of these cells may result in impaired behavior and contribute to sleep disturbance [65]. The regulation of circRNAs in microglia is of particular interest, as these cells are in close contact with synapses and, like neuronal cells, express genes with long introns - a common source of circRNAs [14,20]. In addition to producing circRNAs, microglia can phagocytose circRNAs exported by neurons or astrocytes to the extracellular space. We sequenced freshly isolated microglia from the mouse hippocampus and frontal cortex during the light and dark phases: ZT4 and ZT16 (per time-point 16 replicates, SE 150bp, average sequencing depth: 42 mil.). Our analyses identified 1234 and 1553 distinct circRNAs in the cortex and hippocampus, respectively (Supplementary Table S4). *Cdr1as* was one of the highest expressed circRNAs in microglia (Figure 3D). Despite an overall increase in circularization during the light phase (Figure 3E), the only circRNA that significantly changed its expression in both the cortex and hippocampus was *Cdr1as* (Figure 3F). In Cortex, *Cdr1as* upregulated during the light phase by ~2.6-fold (DESeq2: adj. *p*-value = 1.6 × 10^−7^) and in the hippocampus by 1.8 fold (DESeq2: adj. *p*-value = 0.002).

## 3. Discussion

Our study monitored circRNA expression in the mouse frontal cortex, hippocampus, and suprachiasmatic nucleus in a 12:12 h LD cycle. We characterize *Cdr1as* circRNA as a novel gene associated with light entrainment in the SCN. We found that *Cdr1as* and *Cyrano*, another non-coding RNA involved in miR-7 regulation in the brain, feature oscillatory patterns throughout the LD cycle that replicate the expression pattern of the twin-peak genes. Light-induced regulation of *Cdr1as* was also observed in the hippocampus, but to a lesser extent compared to SCN. Although there is no evidence that photic impulse directly regulates *Cdr1as* expression, we show that genes upregulated upon *Cdr1as* deletion are highly enriched in the light-induced immediate response pathway. Moreover, we demonstrate that the strong regulation of the synaptic twin-peak cluster during the 12 h of the dark cycle (wake phase for mice) occurs only after withdrawal from the light cycle. Pembroke et al. [7] reported that the twin-peak module is highly enriched with genes involved in synaptic transmission, long-term potentiation, calcium signaling, and gated channel activity. On the other hand, Piwecka et al. [31] demonstrated that the loss of *Cdr1as* circRNA in mice results in aberrant excitatory synaptic transmission. In the retina, deletion of *Cdr1as* circRNA results in increased beta-wave amplitude of the photopic electrophysiological response and reduced vision contrast sensitivity [54]. Together, these observations suggest that *Cdr1as* plays an important role in communicating light from the retina to SCN, most likely through the regulation of glutamatergic neurotransmission. Additionally, our results shed more light on the deregulation of certain clock genes in the brain of *Cdr1as* deficient mice, as reported previously [31]. Loss of *Cdr1as* also caused the downregulation of a miRNA family (miR-96/miR-182/miR-183), specifically in the cortical region [31]. Only recently has it been discovered that these miRNAs are involved in the modulation of the circadian rhythm [12]. 

Since two other members of the regulatory network described by Kleaveland et al. [53], miR-7 and *Cyrano*, are also expressed in the SCN, the central circadian pacemaker emerges as a perfect system for studying the interplay of these non-coding molecules. We believe that further experiments exploring the exact pathway by which *Cdr1as* influences the activity of SCN neurons should take into account the oscillatory pattern of *Cdr1as* along with miR-7 targets from the twin-peak module. One surprising observation in the SCN is that *Cdr1as*, a highly stable circular RNA, is downregulated by more than two-fold during 4 h (from ZT14 to ZT18, Supplementary Figure S1). One possible mechanism may be the cleavage of circRNA by miR-671 [66]. In addition, *Cdr1as* turnover may occur due to structure-mediated RNA decay by *Upf1* and *G3bp1* [67]. Both genes are highly expressed in the SCN [7]. We also observed regulation of this abundant circRNA expression throughout the LD cycle in the hippocampus. It is generally accepted that circular RNAs are naturally long-lived in physiological conditions and change their expression significantly only over longer processes (e.g., development, neuronal maturation, or aging) and in pathological conditions (cancer and neurodegeneration). On the contrary, our observations point toward the dynamic nature of circRNA *Cdr1as* in two brain regions. It is interesting to think about the implications of this finding for cancer. *Cdr1as* is deregulated in multiple tumor types [68,69,70,71,72,73,74,75], including breast cancer, where it promotes the metastatic phenotype [76]. In addition, the inhibition of *Cdr1as* increases the sensitivity of drug-resistant breast cancer cells [77]. It was recently shown that the generation of circulating tumor cells in breast cancer does not occur continuously but during the sleep phase [41]. Thus, the characterization of *Cdr1as* expression patterns over the wake-sleep cycle might bring new insights into the metastasis of cancer cells.

Contemplating the importance of these findings for neurodegenerative disorders, the interaction of *Cdr1as*/miR-7 with alpha-synuclein (*Snca*) (twin-peak gene) is particularly interesting. Abnormal expression of *Snca* and its aggregation are critical in the pathophysiology of Parkinson’s disease (PD) [78]. Moreover, in PD patients, miR-7 is significantly downregulated in the brain regions that undergo neurodegeneration during the course of the disease [44]. Due to its binding ability to *Snca* 3′UTR in vivo and subsequent regulation of protein translation, miR-7 is becoming widely appreciated as an important therapeutic target for PD [44,45,51,52]. Sleep disorders are a common feature of patients in the early stages of the disease [79]. Thus, understanding the strong regulation of *Cdr1as* in the central circadian pacemaker may provide a new paradigm for studying the early onset of PD.

It is important to highlight that the present study demonstrates oscillations in circRNA expression patterns in physiological conditions in adult animals. As research is developing around circRNA dynamics in brain development and specific disorders, it will be highly relevant to determine how the latter are affected in disease from a circadian perspective. In preclinical studies, mice are most often sacrificed for downstream molecular analyses during their sleep phase, while in clinical studies, the time of collection of samples from human patients and controls is rarely taken into consideration as a biologically relevant variable. Altered sleep and circadian patterns are, however, hallmarks of psychiatric and neurodegenerative diseases [80,81]. This study provides an example of how modeling circadian variability, both in coding and non-coding RNAs like *Cdr1as*, can widen our understanding of transcriptional dynamics in these conditions. Finally, circRNAs are actively screened as biomarkers in many diseases [38,39]. Our findings suggest that their predictivity may be time sensitive, making it important to carry out these experiments in a time-controlled manner.

## 4. Conclusions

CircRNAs have not only a developmental stage- but also daytime-dependent expression. Specifically, circular RNA *Cdr1as* is regulated throughout light-dark cycles in the SCN, which may influence the organism’s adaptation to quick daily changes.

## 5. Materials and Methods

### 5.1. Animals

Nine- to ten-week-old male C57BL6/N mice (Charles River Laboratories, Sulzfeld, Germany) were used throughout the study. We allocated 3-5 animals per cage in individually ventilated cages (IVCs). The animal vivarium was a specific pathogen-free (SPF) holding room that was temperature- and humidity-controlled (21 ± 3 °C, 50 ± 10%). Animals used for the microglial isolation were kept under a reversed light-dark cycle (lights off 09:00 AM–09.00 PM), while the animals used for whole hippocampal and frontal lobe tissue sequencing were kept on a normal light-dark cycle (lights on 6 AM–6 PM). All animals had ad libitum access to the same food (Kliba 3436, Kaiseraugst, Switzerland) and water throughout the study. All procedures described in the present study had been previously approved by the Cantonal Veterinarian’s Office of Zurich, and all efforts were made to minimize the number of animals used and their suffering.

### 5.2. Brain Dissociation and Cell Isolation

Brain tissue dissociation and microglia cell isolation were performed according to a protocol recently optimized and published by us [82]. The protocol was carried out at 4 °C to avoid cell activation during the isolation procedure. Briefly, the animals were deeply anesthetized with an overdose of Nembutal (Abbott Laboratories, North Chicago, IL, USA) and transcardially perfused with 15 mL ice-cold, calcium- and magnesium-free Dulbecco’s phosphate-buffered saline (DPBS, pH 7.3–7.4). The brains were quickly removed and washed with ice-cold DPBS, after which the hippocampi and frontal cortices were dissected on a cooled petri dish and placed in an ice-cold Hibernate-A medium. Mechanical dissociation at 4 °C was carried out on ice. The tissue was dissociated in 1.5 mL Hibernate-A medium in a 1 mL Dounce homogenizer with a loose pestle. The homogenized tissue was then sieved through a 70 μm cell strainer. The homogenates were pelleted at 450× *g* for 6 min at 4 °C. The supernatants were removed, and the pellets were re-suspended with a P1000 micropipette, applying a pipette-tip cut-off. 500 microliters of freshly prepared isotonic percoll solution was then added to each sample (final volume: 2 mL) and mixed well. Percoll was rendered isotonic by mixing 1 part of 10× calcium- and magnesium-free DPBS (pH 7.3–7.4) with 9-parts of percoll. Importantly, the pH of percoll was adjusted to 7.3–7.4 with 5 molar hydrochloric acid before starting the isolation procedure. The percoll solution was mixed properly with the cell suspension, after which 2 mL of DPBS was gently layered on top of it with a pipette boy set at the slowest speed, creating two separate layers. The samples were centrifuged for 10 min at 3000× *g*. The centrifugation resulted in an upper layer consisting of DPBS and a lower layer consisting of percoll. The two layers were separated by a disk of myelin and debris, while the cells were located at the bottom of the tube. The layers were aspirated, leaving about 500 μL. The cells were then washed once in DPBS and pelleted by centrifuging them at 460× *g* for 10 min at 4 °C. This pellet consists of total brain cells, including microglial cells.

### 5.3. Microglia Cell Magnetic Sorting

Microglia cells were isolated via magnetic-activated cell sorting (MACS) using mouse anti-CD11b magnetic microbeads (Miltenyi Biotec, Bergisch Gladbach, Germany), according to the manufacturer’s instructions, with some modifications. The MACS buffer used consisted of 2% bovine serum albumin (BSA) diluted in DPBS from a 7.5% cell culture-grade BSA stock (Thermo Fisher Scientific Inc., Waltham, MA, USA). Total hippocampal/frontal cortex cell pellets after percoll separation (see above) were re-suspended in 90 μL MACS buffer and 10 μL anti-mouse-CD11b magnetic beads (Miltenyi Biotec, Bergisch Gladbach, Germany). The cells were then incubated for 15 min at 4 °C. Cells were washed with 1 mL MACS buffer and pelleted at 300 rcf for 5 min at 4 °C. The cells were then passed through an MS MACS column (Miltenyi Biotec, Bergisch Gladbach, Germany) attached to a magnet. After washing the columns three times with MACS buffer, microglia were flushed from the column with 1 mL of MACS buffer and pelleted at 300 rcf for 5 min at 4 °C. Cell pellets were then snap-frozen in liquid nitrogen and stored at −80 °C.

### 5.4. Hippocampal and Frontal Lobe Tissue Collection

For the total RNA-seq of whole brain tissue, RNA was extracted from adult male mice total hippocampi and frontal lobes. Briefly, deeply anesthetized adult male mice were intracardially perfused with ice-cold Dulbecco’s phosphate-buffered saline (DPBS) to remove blood. Hippocampi and frontal lobes were dissected immediately afterward in a pre-cooled sterile Petri dish on ice. The brain regions were immediately transferred to an RNAse-free Eppendorf tube, snap-frozen in liquid nitrogen, and stored at −80 °C until RNA extraction.

### 5.5. Total RNA Extraction

Total RNA from freshly isolated microglia and from hippocampal and frontal lobe tissue was extracted via phenol/chloroform extraction using the SPLIT-RNA extraction kit (Lexogen, product code: 008.48), according to the manufacturer’s instructions. The RNA was treated with Turbo DNase I (Ambion, product code AM1907) to remove traces of genomic DNA. Following DNase I treatment, the RNA was stored at −80 °C until library preparation.

### 5.6. Total RNA Library Preparation and Sequencing

Before library preparation, the integrity of each sample was assessed on an Agilent TapeStation system 4150 using RNA screen tape (Agilent Technologies Inc., Santa Clara, CA, USA). In contrast, RNA concentrations were measured using a Qubit 4 fluorometer (ThermoFisher Scientific Inc., Waltham, MA, USA). 100 nanograms of total RNA was used as input for ribosomal RNA (rRNA) depletion using the NEBNext rRNA depletion kit (New England BioLabs Inc., Ipswich, MA, USA, product code: E6350), according to the manufacturer’s instructions. Following rRNA depletion, total RNA libraries were built using the NEBNext Ultra II library prep kit from Illumina (New England BioLabs Inc., Ipswich, MA, USA, product code: E7775), according to the manufacturer’s instructions. The yield of amplified libraries was measured on a Qubit 4 fluorometer using a Qubit high-sensitivity DNA kit (HS DNA kit). Amplified libraries were further analyzed on HS D1000 screen tape on a TapeStation system 4150 to assess library size and molarity prior to pooling. The libraries were sequenced using Illumina HISeq-4000.

### 5.7. Bioinformatic Methods

For circRNA detection, total RNA seq reads were mapped to the mouse GRCm38 genome with BWA [83] (version 0.7.17-r1188) using the -T 19 option. CircRNAs were identified using CIRI2 [84] with default parameters. For further analyses, we used circRNAs identified in at least two replicates at the same time point. To compare circRNAs with circbase datasets, circbase coordinates were translated from mm9 to mm10 genomes using the UCSC liftOver tool. To calculate the *p*-values and log2 fold changes for circRNA expression changes, we used the DESeq2 [85] (version 1.22.1) package (normalization: rld, test: Wald’s test, *p*-value adjustment: Benjamini-Hochberg). The circRNA expression table was concatenated with the linear constitutive gene expression table. To calculate the expression of the linear genes, we mapped the reads to the mouse GRCm38 genome with STAR (version 2.7.3.a, default parameters, [86]) and assigned reads to genes with featureCounts [87] (version 2.0.0). For the FPKM calculation, the read counts were normalized to the total number of uniquely mapped reads per sample and the length of the genes, as reported by featureCounts (calculated from the gencode GRCm38 vM12 GTF file). To compare the linear and circular RNA expression changes in the hippocampus and cortex, we quantified the sum of all spliced reads mapped to the gene (featureCounts -J parameter). The evidence plots for Figure 2A,B were produced with R tmod (version 0.46.2 [88]). Gene ontology analysis for Supplementary Table S2 and Figure 2E was carried out with R tmod and msigdbr (7.4.1) packages.

## Data Availability

The raw sequencing data have been deposited in GEO under accession numbers GSE199791 and GSE200314 and will be available upon publication. The R code for data analyses and figures can be downloaded from github.com/bihealth/circ_circ (accessed date: 31 August 2022).

## Supplementary Materials

The following supporting information can be downloaded at: https://www.mdpi.com/article/10.3390/ijms232012347/s1.
