# Supplementary figures and images for: Analyses of circRNA Expression throughout the Light-Dark Cycle Reveal a Strong Regulation of Cdr1as, Associated with Light Entrainment in the SCN
Source: Int J Mol Sci. 2022 Oct 15;23(20):12347. doi: 10.3390/ijms232012347 (PMC9604060; doi:10.3390/ijms232012347)

Supplementary Figure S1

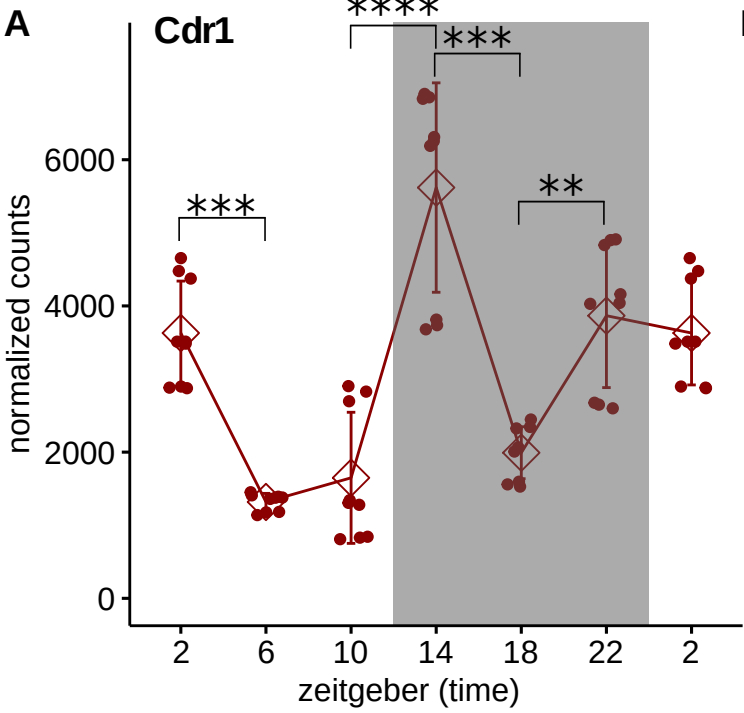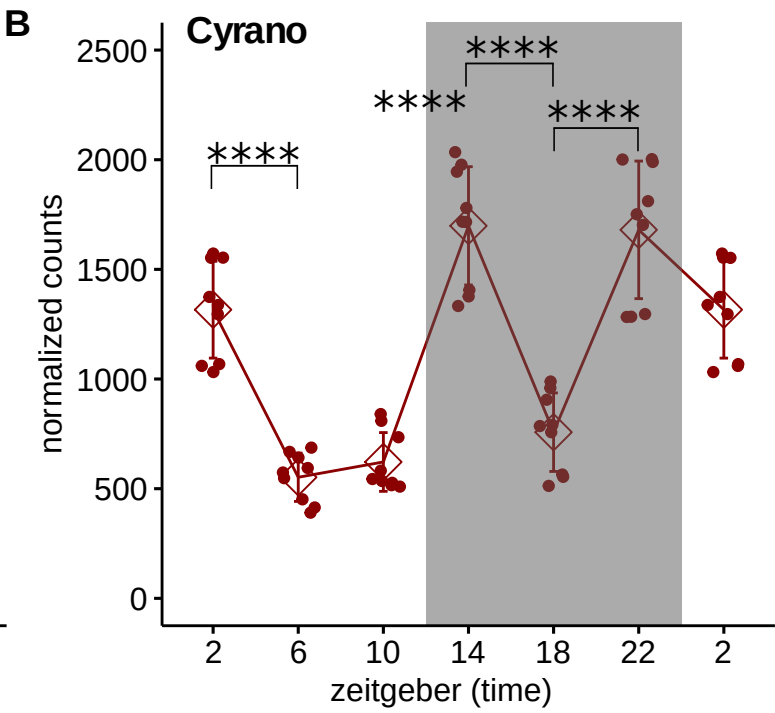

## Supplementary Figure S2

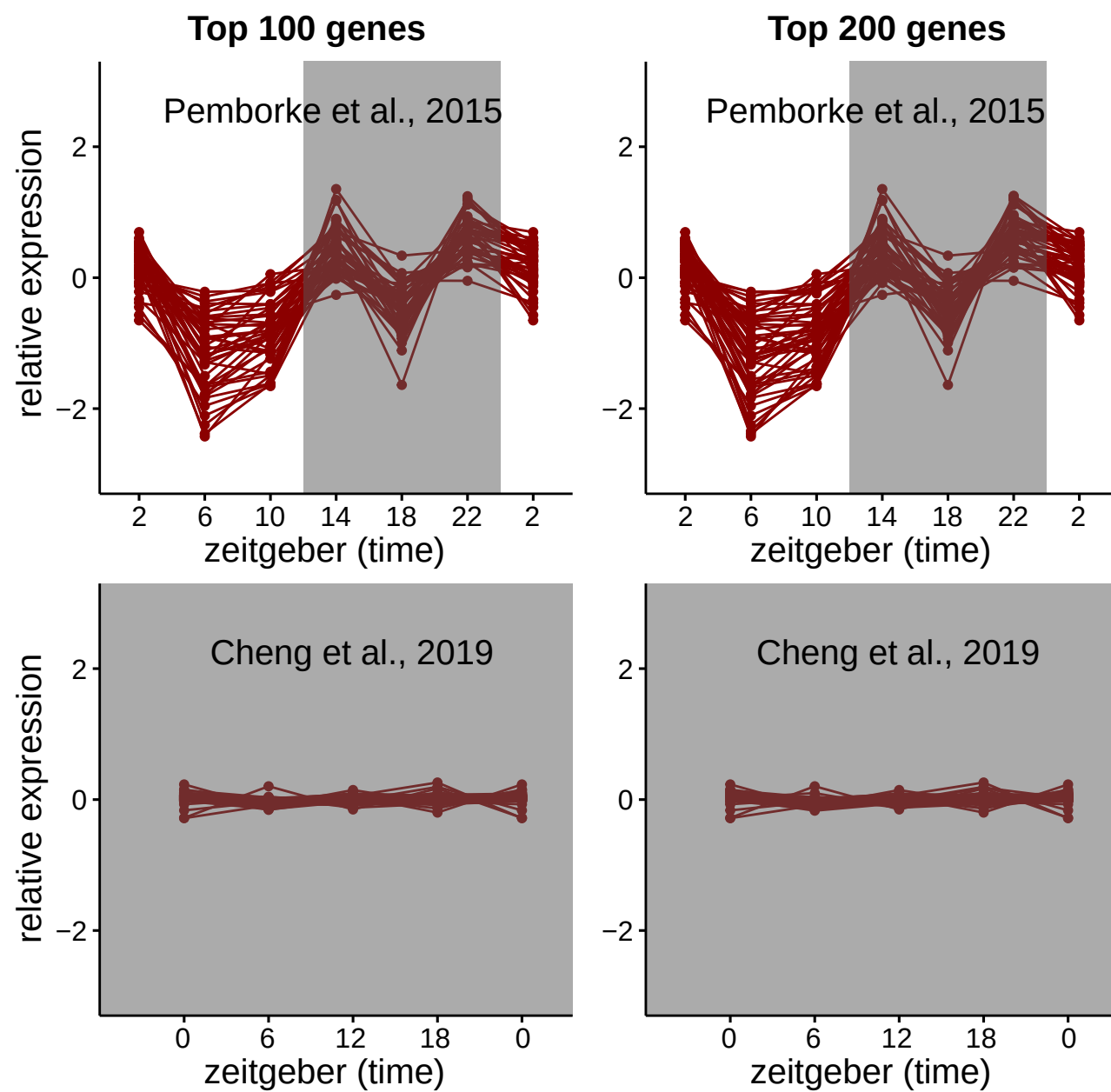

# Supplementary Figure S3

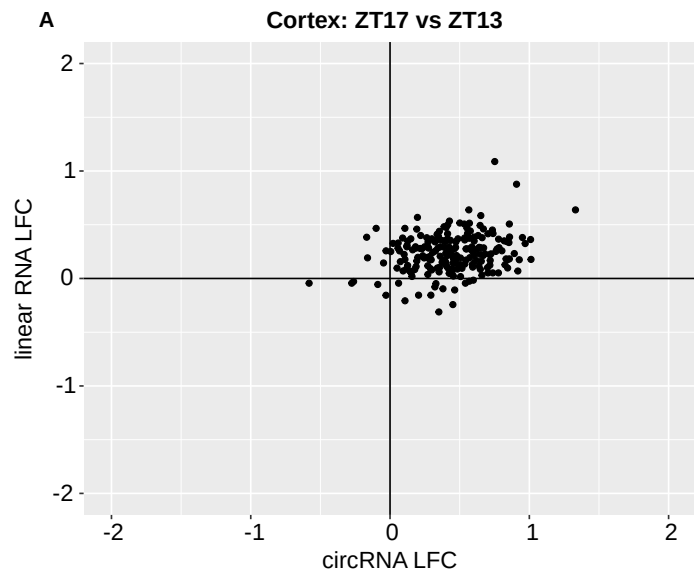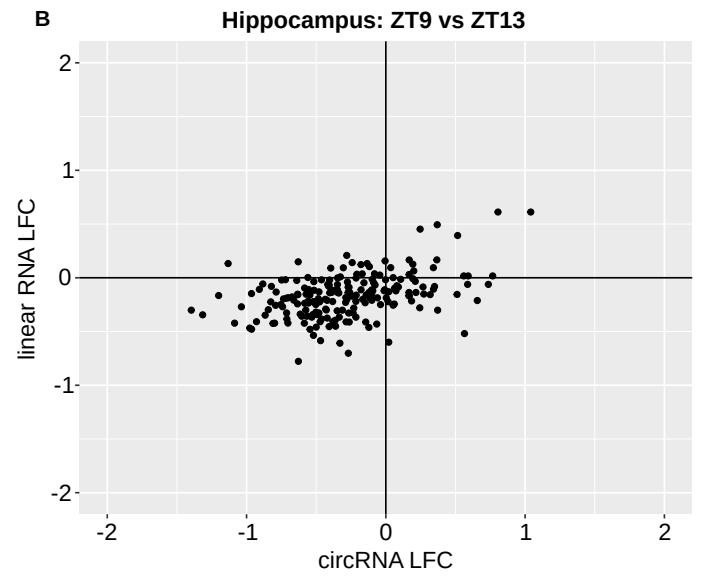

Supplement: Supplementary file 1 [file ijms-23-12347-s001.zip › Supplementary_Figures.pdf]
